# Infiltrating circulating monocytes provide an important source of BMP4 at the early stage of spinal cord injury

**DOI:** 10.1242/dmm.049856

**Published:** 2023-01-18

**Authors:** Weiyun Shen, Shuxin Liu, Xiaojing Wei, Yaping Wang, Lin Yang

**Affiliations:** ^1^ Department of Anesthesiology, The Second Xiangya Hospital, Central South University, Changsha 41000, Hunan Province, China; ^2^ Hunan Province Center for Clinical Anesthesia and Anesthesiology, Research Institute of Central South University, Changsha 41000, Hunan Province, China; ^3^ Department of Pain Management, The Second Xiangya Hospital, Central South University, Changsha 41000, China

**Keywords:** Bone morphogenetic protein 4, Monocyte, Monocyte-derived macrophage, Spinal cord injury, Blood spinal cord barrier

## Abstract

Bone morphogenetic protein (BMP)4 plays a critical role in regulating neuronal and glial activity in the course of spinal cord injury (SCI). The underlying cause and cellular source of BMP4 accumulation at the injured spinal cord remain unclear. Here, we observed that plasma BMP4 levels are statistically higher in SCI patients than in healthy donors. When comparing rats in the sham group (T9 laminectomy without SCI) with rats in the SCI group, we found a persistent decline in BBB scores, together with necrosis and mononuclear cell accumulation at the contusion site. Moreover, during 2 weeks after SCI both plasma and cerebrospinal fluid levels of BMP4 displayed notable elevation, and a positive correlation. Importantly, percentages of circulating BMP4-positive (BMP4^+^) monocytes and infiltrating MDMs were higher in the SCI group than in the sham group. Finally, in the SCI+clodronate liposome group, depletion of monocytes effectively attenuated the accumulation of both BMP4^+^ MDMs and BMP4 in the injured spinal cord. Our results indicated that, following SCI, infiltrating MDMs provide an important source of BMP4 in the injured spinal cord and, therefore, might serve as a potential therapeutic target.

## Teaser

**Summary:** Following a spinal cord injury, circulating monocytes highly express BMP4 and increase the infiltration of monocyte-derived macrophages, also with upregulated BMP4 expression, into the injured spinal cord.

## INTRODUCTION

Spinal cord injury (SCI) is a devastating complication after traumatic or non-traumatic damage insulting the spinal cord. SCI patients often suffer from long-term disability and paralysis (McDonald and Sadowsky, 2002). Once the injury has manifested clinically, conventional treatment measures, including early surgical decompression and high-dose corticosteroid therapy, only provide limited therapeutic effect (Karsy and Hawryluk, 2019). One prominent pathological feature of SCI is breakdown of the blood–spinal cord barrier (BSCB), which alters the composition of the microenvironment of the central nervous system (CNS) by allowing the invasion of circulating signals, especially monocytes, into the injured spinal cord (Diaz et al., 2021; Gao et al., 2021; Döring et al., 2015; Heller et al., 2021). A growing body of evidence (Döring et al., 2015; Heller et al., 2021; Durafourt et al., 2012; Girard et al., 2013; Yamasaki et al., 2014) has demonstrated that infiltrating monocyte-derived macrophages (MDMs) play a critical role in the regulation of inflammation and nerve regeneration, which might significantly influence neurological recovery. Thus, therapeutic strategies targeting the invasion of circulating monocytes should be addressed for the treatment of SCI.

As a member of the transforming growth factor-β (TGF-β) superfamily, bone morphogenetic protein (BMP)4 serves as an important regulator of tissue homeostasis and cell differentiation for both the developmental CNS (Polevoy et al., 2019; Miller et al., 2004) and adult CNS post-SCI (Cole et al., 2016; Al-Sammarraie and Ray, 2021; Wang et al., 2014). Strong evidence has revealed that SCI is accompanied by the upregulation of endogenous BMP4, which further induces glial scar formation (Xiao et al., 2010; Fuller et al., 2007), demyelination (Wang et al., 2011) and gradual loss of neurons nearby (Hart et al., 2020). Furthermore, antagonizing the BMP4 signaling cascade achieves the promotion of both remyelination and neurological improvement (Petersen et al., 2021; Lei et al., 2012; Matsuura et al., 2008), adding to the evidence that BMP4 mainly plays a detrimental role in the process of SCI. Although the actions of BMP4 have largely been elucidated, the underlying cause and cellular source of BMP4 accumulation in the injured spinal cord remain unknown. Notably, an *in vitro* study (Jo et al., 2021) revealed that human monocytes express BMP4 under a specific stimulus. In addition, in our recent clinical study (Zhao et al., 2021), we reported that, during surgery, patients' circulating BMP4 levels significantly increase and display a close relationship with inflammation severity. In light of the evidence above, we further hypothesize that, after SCI, BMP4 is upregulated in circulating monocytes, which further transfer to the injured spinal cord through the disrupted BSCB, therefore contributing to the BMP4 upregulation at the lesion site.

In the present study, we tested the BMP4 levels of circulating monocytes in blood and in infiltrating MDMs in the spinal cord following an SCI. In addition, we tested whether depletion of circulating monocytes could attenuate BMP4 upregulation in the injured spinal cord. Our findings increase understanding of the cellular source of BMP4 accumulation at the lesion site, which could help with identifying new approaches for treating SCI patients.

## RESULTS

### BMP4 is upregulated in patients with SCI

**Fig. 1.**
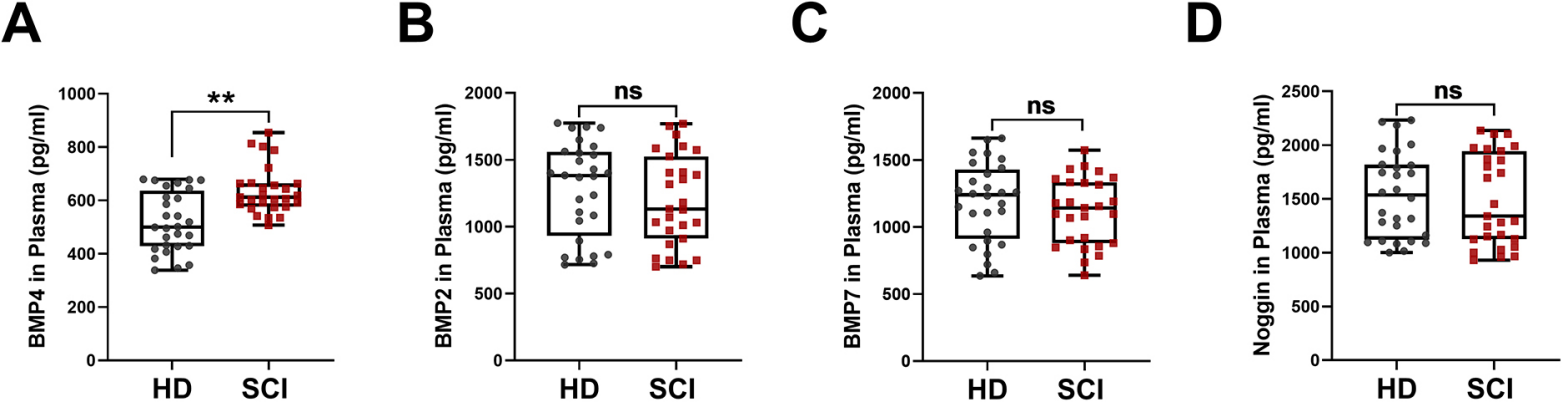
**Plasma levels of BMP2/4/7 and noggin in spinal cord injury (SCI) patients and healthy donors.** (A-D) Enzyme-linked immunosorbent assay (ELISA) was used to detect the plasma concentrations of BMP4 (A), BMP2 (B), BMP7 (C) and noggin (D) in SCI patients and healthy donors (HD). Mann–Whitney *U*-tests were performed. **P<0.01. ns, not significant.

Clinical data, including patients' general information, American Spinal Injury Association (ASIA) impairment scale grade, and plasma levels of BMP2/4/7 and noggin, are provided in Table 1 and Fig. 1. Compared with the levels in the healthy donor group [500.20 (429.50–620.34); median (interquartile range)], we observed markedly increased BMP4 plasma levels in the SCI group [611.96 (580.69–663.09) pg/ml, *P*<0.01; Fig. 1A], whereas BMP2 [1378.37 (969.21–1543.66) versus 1132.03 (941.50–1466.10) pg/ml, *P*=0.37; Fig. 1B], BMP7 [1240.52 (944.87–1407.51) versus 1143.02 (890.71–1330.97) pg/ml, *P*=0.35; Fig. 1C] and noggin [1510.14 (1135.49–1816.52) versus 1339.98 (1125.85–1907.88) pg/ml, *P*=0.67; Fig. 1D] plasma levels were comparable between the two groups. These results suggested that patients' circulating BMP4 are elevated after an SCI.

**Table 1.**
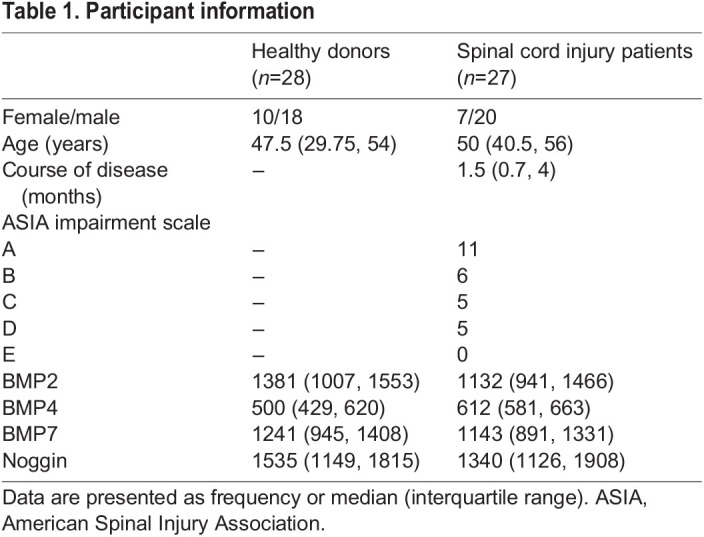
Participant information

### SCI causes persistent paraplegia, nerve injury and cell loss in the injured spinal cord

**Fig. 2.**
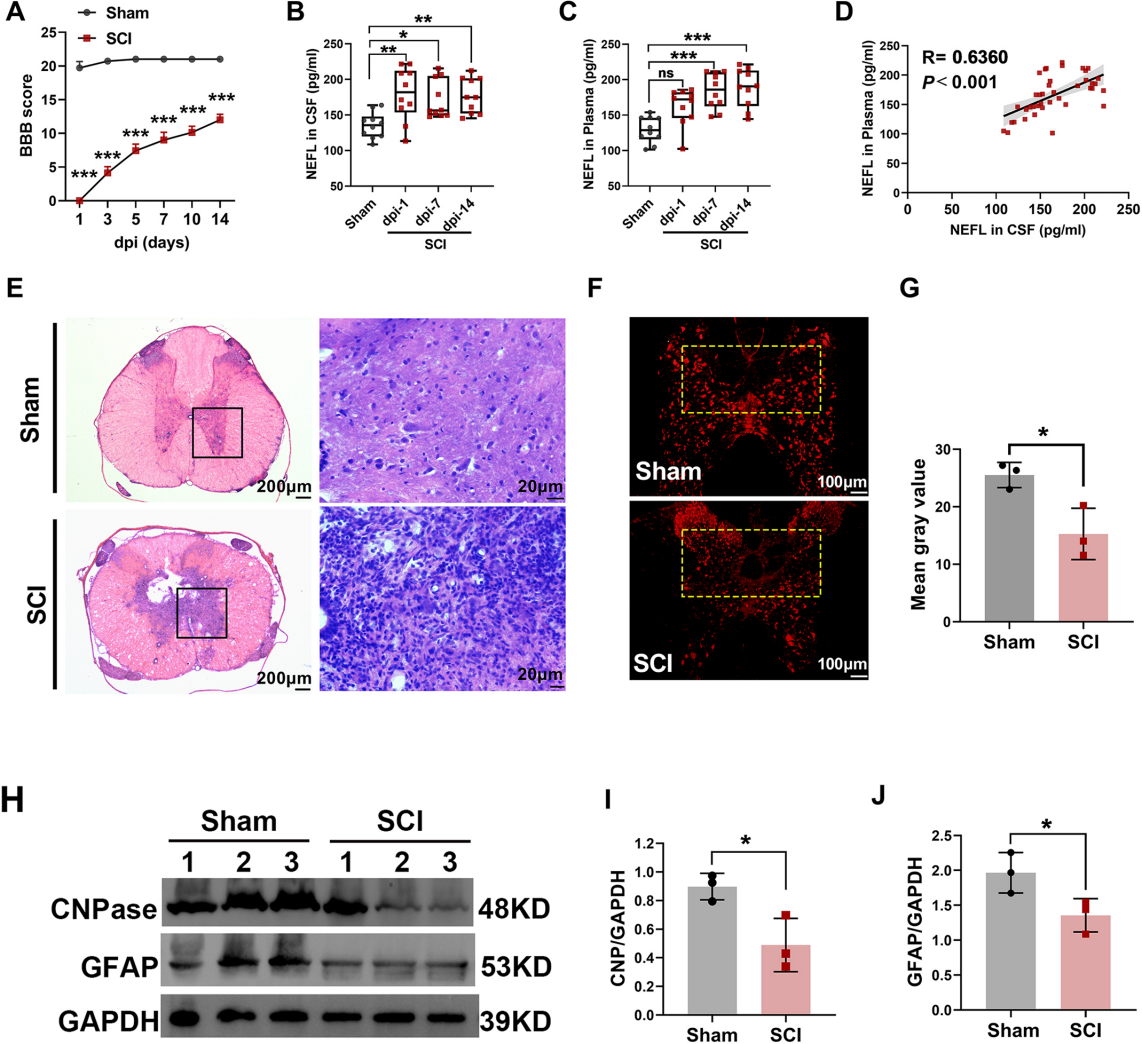
**SCI causes persistent paraplegia, nerve injury and cell loss in the injured spinal cord.** Rats were subjected to sham or SCI interventions. (A-C) Then, for both groups, motor function was evaluated by Basso-Beattie-Bresnahan (BBB) scale within the consecutive 14 days (A); and, at day post injury (dpi)-3, cerebrospinal fluid (CSF) (B) and plasma (C) were harvested and assayed by ELISA to detect the NEFL concentrations (B,C). (D) Correlation in NEFL levels between CSF and plasma. (E) At dpi-3, frozen sections of spinal cord tissue were harvested and stained with Hematoxylin and Eosin (H&E) to show the morphological changes. (F,G) Immunofluorescence was performed to show expression changes in NEUN (neuron marker, red) at the lesion site of the spinal cord (in the yellow dashed line box) (F) and quantified (G). (H-J) Western blotting was employed to detect the expression changes in CNPase (oligodendrocyte marker) and GFAP (astrocyte marker) in the spinal cord (H) and quantified (CNPase, I; GFAP, J). Unpaired two-tailed Student's *t*-tests (G,I,J), Kruskal–Wallis test followed by Dunn's multiple tests (B,C), two-way ANOVA followed by Sidak's multiple comparison tests (A) and Spearman's rank correlation analysis (D) were performed. *P<0.05, **P<0.01 and ***P<0.001. ns, not significant.

Experimental rats were divided into three groups for evaluation: (1) sham (rats that underwent a T9 laminectomy without SCI); (2) SCI (rats that underwent a T9 laminectomy, together with SCI induced by falling objects); and (3) SCI+clodronate liposome (CLL) [rats that received CLL intra-caudal vein administration 3 days before and immediately after the SCI intervention]. After the establishment of the SCI model, the Basso-Beattie-Bresnahan (BBB) scores of rats in the sham group were maintained at the baseline of 21; rats with SCI developed significantly lower scores, which initiated at day post injury (dpi)-1 (totally disabled, with BBB scores of 0, *P*<0.001) and lasted until dpi-14 [11.86±0.90 (mean±s.d.), *P*<0.001] (Fig. 2A), indicating that SCI induced long-term paraplegia for at least 2 weeks.

Neurofilament (NEFL) is a stable and exclusive structural protein of the neuronal axon. Previous studies have demonstrated that, upon nerve injury, NEFL is released from the cerebrospinal fluid (CSF) to the bloodstream, thus making circulating NEFL a reliable biomarker to predict SCI severity (Butryn et al., 2020; Kuhle et al., 2015; Barro et al., 2018; Dang Do et al., 2021). In the present study, the plasma levels of NEFL in the SCI group were comparable with those in the sham group [128.78 (119.65–145.91) pg/ml] at dpi-1 [170.08 (150.52–180.74) pg/ml, *P*=0.08], then increased sharply at dpi-7 [185.97 (166.82–208.45) pg/ml, 1.44-fold, *P*<0.001] and dpi-14 [190.51 (171.19–210.77) pg/ml, 1.48-fold, *P*<0.001] (Fig. 2B). Meanwhile, the CSF levels of NEFL in the SCI group showed sustained elevations at dpi-1 [181.84 (161.28–208.84) pg/ml, 1.34-fold, *P*<0.01], dpi-7 [156.23 (150.30–197.82) pg/ml, 1.15-fold, *P*<0.05] and dpi-14 [174.83 (154.66–200.58) pg/ml, 1.29-fold, *P*<0.01] compared with the levels in the sham group [135.61 (121.50–143.31) pg/ml] (Fig. 2C). Additionally, a strong positive correlation (*P*<0.001, *R*=0.64) existed between central (CSF) and peripheral (serum) NEFL (Fig. 2D). The plasma NEFL elevations and correlation between CSF and blood in the present study further indicate that neuroaxonal injury persisted throughout the 2 weeks after the SCI intervention.

Then, at dpi-3, Hematoxylin and Eosin (H&E) staining of the spinal tissue revealed an evident necrotic cavity formation located at the contusion site of the spinal cord in rats of the SCI group, accompanied by evidently increasing diffusion of mononuclear cells in the gray matter (Fig. 2E). At the same time point, immunofluorescence revealed that SCI intervention caused a significant reduction in the number of NEUN^+^ (also known as RBFOX3^+^) cells (neurons) at the lesion area (marked in the yellow dashed line box, *P*<0.05; Fig. 2F,G). Western blotting results further showed that, compared with the levels in the sham group, the levels of expression of GFAP (a marker of astrocytes) and CNPase (a marker of oligodendrocytes) in the SCI group were downregulated 0.69-fold (*P*<0.05; Fig. 2H,I) and 0.54-fold (*P*<0.05; Fig. 2H,J).

All the above results confirmed the successful establishment of SCI, characterized by neurological dysfunction, nerve injury and cell loss in the early phase of the disease.

### SCI induces BMP4 upregulation in circulating monocytes

**Fig. 3.**
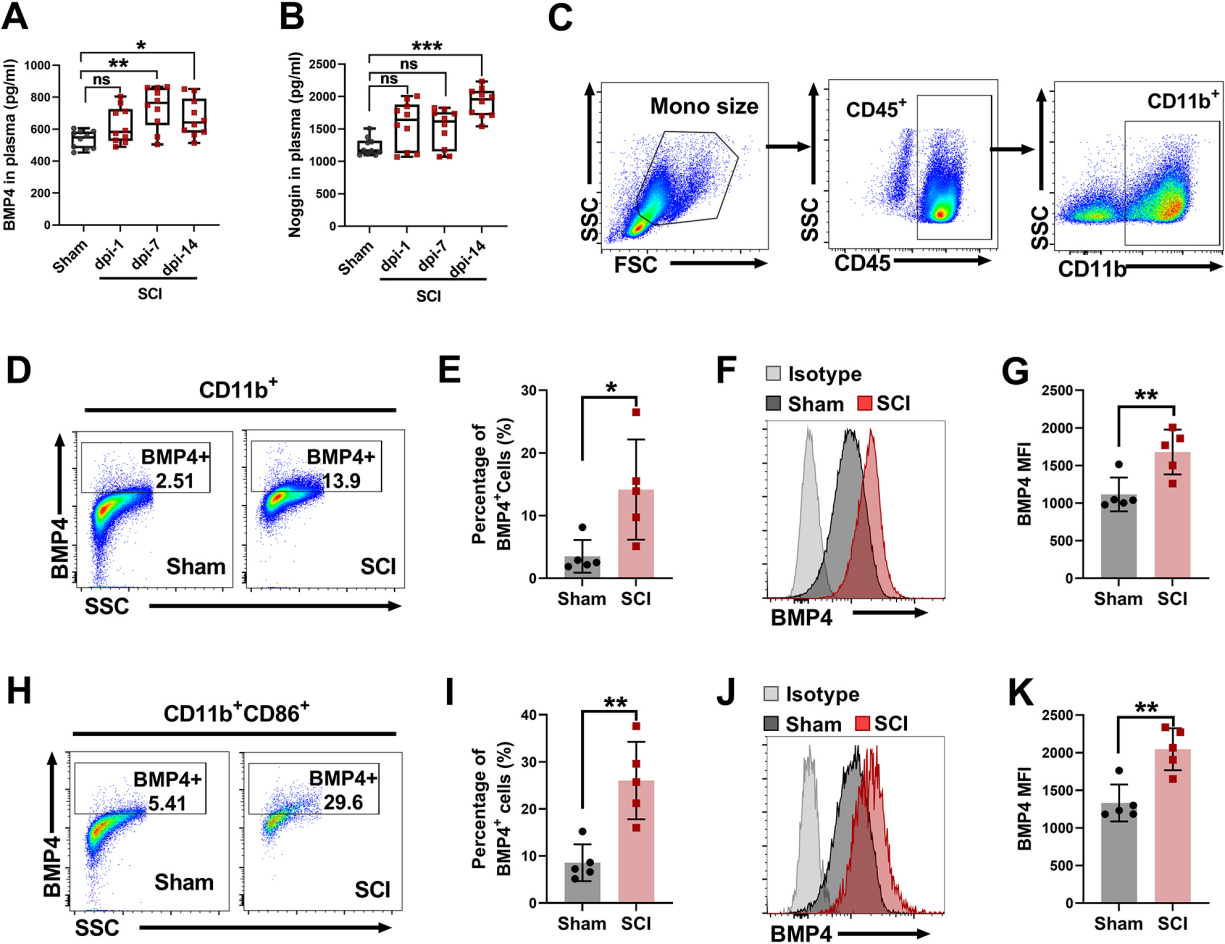
**SCI induces BMP4 upregulation in circulating monocytes.** Blood was collected from SCI and sham rats for ELISA and cytometry analysis. (A,B) ELISA was conducted to detect the BMP4 (A) and noggin (B) levels in the plasma at dpi-1, dpi-7 and dpi-14. (C-K) Peripheral blood mononuclear cells (PBMCs) were isolated from the whole blood by Ficoll gradient separation and analyzed by flow cytometry at dpi-3. (C) The steps and gating strategies of rat circulating monocytes (CD45^+^CD11b^+^) from the PBMCs. (D,E) Representative flow cytometry plots (D) and statistical data (E) showing the percentage of BMP4^+^ cells in circulating monocytes. (F,G) Flow cytometric histogram (F) and statistical data (G) showing the mean fluorescence intensity (MFI) of BMP4 expression in circulating monocytes. (H,I) Representative flow cytometry plots (H) and statistical data (I) showing the percentage of BMP4^+^ cells out of CD86^+^ monocytes. (J,K) Flow cytometric histogram (J) and statistical data (K) showing BMP4 MFI in CD86^+^ monocytes. Unpaired two-tailed Student's *t*-tests (E,G,I,K) and Kruskal–Wallis test followed by Dunn's multiple tests (A,B) were performed. *P<0.05, **P<0.01 and ***P<0.001. ns, not significant. FSC, forward scatter; SSC, side scatter.

First, circulating BMP4 levels in the experimental rats were tested using enzyme-linked immunosorbent assay (ELISA). Similar to the clinical findings, the results showed that the BMP4 plasma levels in the SCI group were comparable with those in the sham group [548.84 (482.93–581.31) pg/ml] at dpi-1 [582.87 (535.42–709.33) pg/ml], then significantly increased by 1.39-fold at dpi-7 [764.34 (668.66–851.17) pg/ml, *P*<0.01] and 1.17-fold at dpi-14 [641.79 (584.90–756.63) pg/ml, *P*<0.05] (Fig. 3A). Plasma levels of noggin in the SCI group remained comparable with those in the sham group [1166.23 (1123.17–1299.64) pg/ml] at dpi-1 [1640.00 (1226.66–1831.76) pg/ml, *P*<0.15] and dpi-7 [1615.20 (1243.13–1741.73) pg/ml, *P*=0.32], then increased by 1.68-fold at dpi-14 [1957.75 (1724.40–2064.82) pg/ml, *P*<0.001] (Fig. 3B).

Second, the expression pattern of monocyte-derived BMP4 was further investigated using flow cytometry. Fig. 3C shows the strategy for detecting the circulating monocytes. As shown in Fig. 3D,E, the percentage of BMP4-positive (BMP4^+^) monocytes at postnatal day (P)3was 3.51±2.61% in the sham group but 14.15±7.99% in the SCI group (*P*<0.05). At the same time point, the mean fluorescence intensity (MFI) of BMP4 in circulating monocytes was markedly higher in the SCI group than in the sham group (1678.40±298.20 versus 1115.40±225.08, *P*<0.01; Fig. 3F,G). Additionally, CD86 was evaluated as a marker of activated monocytes. The results further showed that the percentage of BMP4^+^ cells in CD86^+^ monocytes was significantly higher in the SCI group than in the sham group (26.02±8.23% versus 8.56±3.91%, *P*<0.01; Fig. 3H,I). The same trends were also detected for MFI of BMP4 in circulating CD86^+^ monocytes (2045.60±279.39 versus 1331.60±245.18, *P*<0.01; Fig. 3J,K).

All the above results indicated that, following SCI, circulating monocytes are activated and contribute to the elevation of plasma BMP4 concentration.

### Infiltrating monocytes contribute to BMP4 accumulation in the injured spinal cord

**Fig. 4.**
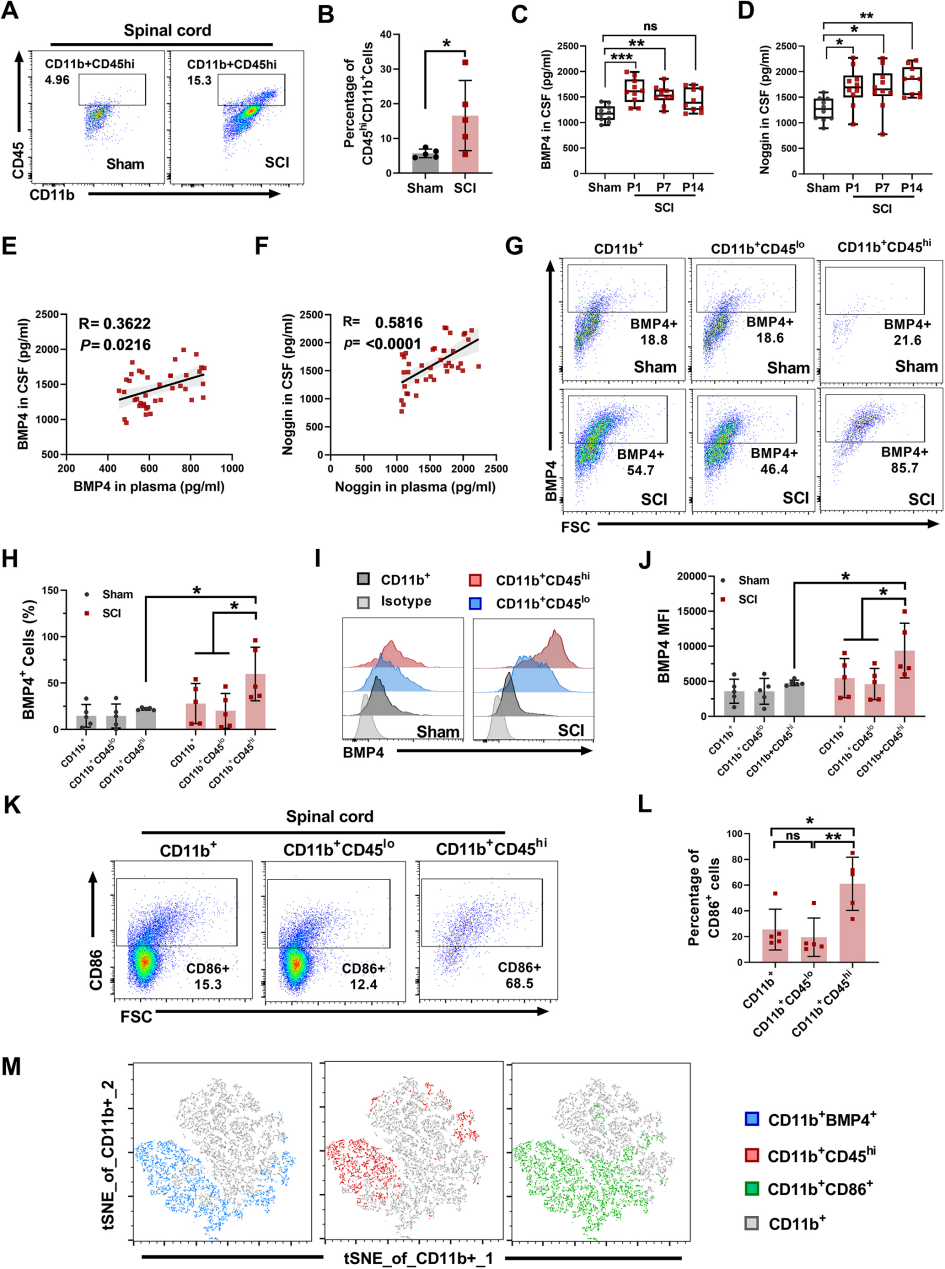
**BMP4 is upregulated in infiltrating monocyte-derived macrophages (MDMs) in the injured spinal cord.** After SCI, spinal cord and CSF were collected from SCI and sham rats for further experiments. (A-D) For flow cytometric analysis, spinal cords were harvested and prepared into a single-cell suspension, and then spinal mononuclear cells were isolated by Percoll gradient centrifugation at 3 days after SCI. (A) Representative flow cytometry plots of CD11b^+^CD45^hi^ (MDMs) cells in SCI and sham groups. Cells previously gated as CD11b^+^. (B) Percentage of CD11b^+^CD45^hi^ (MDMs) cells in spinal CD11b^+^ cells. (C,D) Levels of BMP4 (C) and noggin (D) in CSF were assayed by ELISA at 1, 7 and 14 days after SCI. (E) Correlation analysis of BMP4 concentration between CSF and serum. (F) Correlation analysis of noggin concentration between CSF and serum. (G) Representative flow cytometry plots of BMP4^+^ cells in each subpopulation of spinal CD11b^+^ cells (CD11b^+^, macrophages/microglia; CD11b^+^CD45^lo^, macroglia; CD11b^+^CD45^hi^, macrophages) from the sham (top row) and SCI (bottom row) groups. (H) Percentage of BMP4^+^ cells in each subpopulation of spinal CD11b^+^ cells from the sham and SCI groups. (I,J) Flow cytometric analysis showing the BMP4 MFI in each subpopulation of spinal CD11b^+^ cells from the SCI and sham groups. Data are presented as a representative flow cytometric histogram (I) and summary graph (J). (K,L) Flow cytometry was performed to examine the percentage of CD86^+^ in each subpopulation of spinal CD11b^+^ cells in SCI rats. Data are presented as a representative flow plot (K) and summary graph (L). (M) t-distributed stochastic neighbor embedding (tSNE) plot of flow cytometry data showing cell–subset distributions (CD11b^+^BMP4^+^, CD11b^+^CD45^hi^ and CD86^+^CD11b^+^ cells) in SCI rats. Data are shown as mean±s.d. Unpaired two-tailed Student's *t*-tests (B), one-way ANOVA followed by Sidak's multiple comparison tests (C,D,H,J,L) and Spearman correlation analysis (E,F) were performed. *P<0.05, **P<0.01 and ***P<0.001. ns, not significant.

First, anti-CD11b (also known as ITGAM) and anti-CD45 (also known as PTPRC) antibodies were used to distinguish the infiltrating MDMs (CD11b^+^CD45^hi^) from microglia (CD11b^+^CD45^lo^) according to previously described methods (Donat et al., 2017; Sousa et al., 2018; Xin et al., 2020; Azambuja et al., 2020) (Fig. S1). As shown in Fig. 4A,B, the percentage of CD11b^+^CD45^hi^ cells (MDMs) in rats of the SCI group (16.58±10.11%) was markedly higher than that in rats of the sham group (5.67±1.23%) at dpi-3 (*P*<0.05), indicating enhancement of the influx of circulating monocytes after SCI took place.

Second, ELISA results showed that, compared with the CSF levels in the sham group (1178.04±156.95 pg/ml), the CSF levels of BMP4 in rats in the SCI group were significantly higher at dpi-1 (1622.20±246.28 pg/ml, *P*<0.001) and dpi-7 (1536.22±181.66 pg/ml, *P*<0.01), but were not significantly different at dpi-14 (1448.33±228.65, *P*=0.06) (Fig. 4C). Meanwhile, the CSF levels of noggin in the SCI group were significantly higher than those in the sham group (1261.24±225.19 pg/ml) at dpi-1 (1696.04±373.47 pg/ml, *P*<0.05), dip-7 (1673.24±421.23 pg/ml, *P*<0.05) and dpi-14 (1817.24±275.36 pg/ml, *P*<0.01) (Fig. 4D). In addition, positive correlations in the levels of BMP4 (*P*<0.05, *R*=0.36, Fig. 4E) and noggin (*P*<0.01, *R*=0.58, Fig. 4F) were found between the plasma and CSF.

Third, cytometric results showed that, at dpi-3, the percentage of BMP4^+^ cells in MDMs (CD11b^+^CD45^hi^) was 59.78±28.89%, which was significantly higher than that in microglia (CD11b^+^CD45^lo^, 20.16±18.66%, *P*<0.05) and total macrophages (CD11b^+^, 27.98±21.61%, *P*<0.05) in the SCI group, and also higher than that in MDMs in the sham group (22.13±1.51%, *P*<0.05) (Fig. 4G,H). Similarly, the MFI of BMP4 in infiltrating MDMs (9389.20±3894.67) was higher than that in microglia (4615.40±2229.88, *P*<0.05) and total macrophages (5463±2790.97, *P*<0.05) in the SCI group, and also higher than that in the sham group (4882.50±377.66, *P*<0.05) (Fig. 4I,J). Next, in the SCI group, the percentage of CD86^+^ cells in MDMs was 57.57±20.34%, which was markedly higher than that in microglia (19.18±13.39%, *P*<0.01) and total macrophages/microglia (CD11b^+^, 27.73±14.35%, *P*<0.05) (Fig. 4K,L). Particularly, as shown in Fig. 4M, the t-distributed stochastic neighbor embedding (tSNE) plot visualizing cell–subset distributions further revealed that, in total macrophages/microglia (CD11b^+^, gray), the infiltrating MDMs (CD11b^+^CD45^hi^ cell subtype, red) were highly BMP4^+^ (blue) and CD86^+^ (green).

Taken together, these results indicate that, after SCI, there is an influx of circulating monocytes, which abundantly express BMP4, into the spinal cord, where they are likely to participate in the crosstalk between the circulatory system and the CNS.

### Depletion of circulating monocytes decreases BMP4 expression in the injured spinal cord

**Fig. 5.**
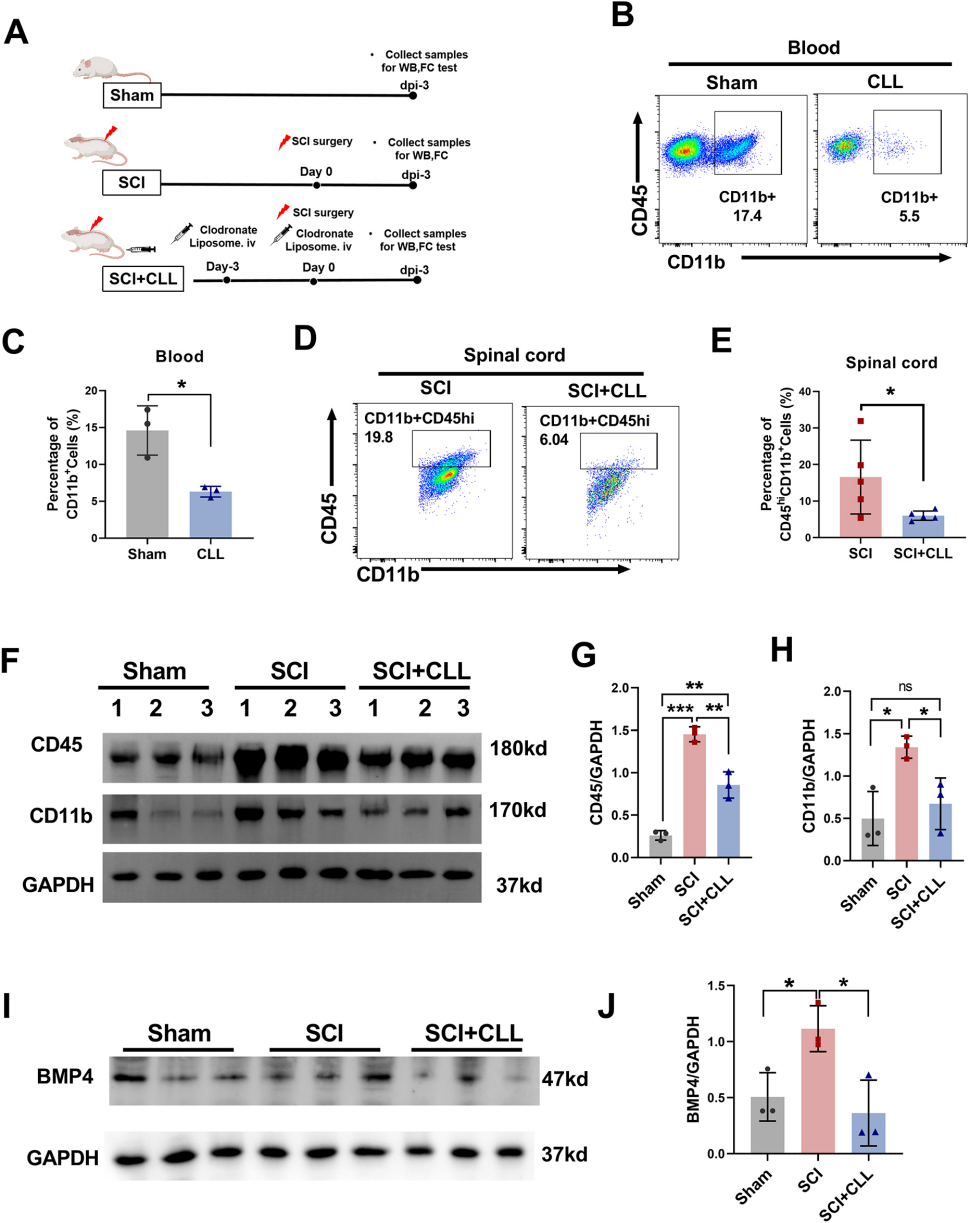
**Depletion of circulating monocytes decreases infiltration of monocytes and BMP4 levels in spinal cord, and alleviates the neurological deficit induced by SCI.** (A) Experimental diagram showing the timeline of drug treatments, SCI surgery and further experiments. (B) Flow cytometry plot showing the efficiency of monocyte clearance in blood at 3 days after injection of clodronate liposome (CLL). (C) Statistical analysis of the monocyte population in blood at 3 days after administration of CLL. (D,E) Flow cytometry analysis showing the percentage of infiltrating MDMs in spinal cord at 3 days after SCI treatment. Data are presented as a representative flow cytometry plot (D) and statistical graph (E). (F) Representative western blot images showing CD45 and CD11b expression in the spinal cord. (G) Gray value ratio of CD45 to GAPDH in spinal cord. (H) Gray value ratio of CD11b to GAPDH in spinal cord. (I,J) The level of BMP4 in spinal cord was assessed by western blotting. Data are presented as representative images (I) and a graph showing the gray value ratio of BMP4 to GAPDH (J). Unpaired two-tailed Student's *t*-tests (C,E) and one-way ANOVA followed by Sidak's multiple comparison tests (G,H,J) were performed. *P<0.05, **P<0.01 and ***P<0.001. ns, not significant. FC, flow cytometry; WB, western blotting.

Upon the above findings that infiltrating MDMs highly express BMP4 following an SCI, we further hypothesized that depletion of circulating monocytes should be able to attenuate MDM infiltration, thus effectively decreasing BMP4 accumulation in the injured spinal cord. In this scenario, CLL was administered to scavenge the circulating monocytes. First, the efficacy of circulating monocyte depletion was confirmed by flow cytometric analyses, in which CLL significantly reduced the percentage of monocytes (CD11b^+^CD45^+^) from 16.45±1.34% (sham level) to 6.00±0.70% at 3 days after intra-caudal vein administration (*P*<0.05; Fig. 5B,C).

Second, the SCI+CLL group showed a significantly lower percentage of infiltrating MDMs (CD11b^+^CD45^hi^) than that of the SCI group (6.01±1.28% versus 16.58±10.11% at dpi-3, *P*<0.05; Fig. 5D,E). Western blotting results further showed markedly elevated CD45 and CD11b expression in the SCI group compared with that in the sham group (CD45, 5.58-fold; CD11b, 2.69-fold; *P*<0.01), which significantly decreased after CLL administration in the SCI+CLL group (CD45, 0.59-fold, *P*<0.05; CD11b, 0.50-fold, *P*<0.05) (Fig. 5F-H). These results indicated that CLL treatment successfully reduced MDM infiltration to the injured spinal cord.

Moreover, western blotting results further showed significantly higher BMP4 expression in rats in the SCI group than in rats in the sham group (2.20-fold, *P*<0.05). Meanwhile, significantly lower BMP4 expression was observed in rats in the SCI+CLL group than in rats in the SCI group (0.33-fold, *P*<0.05), indicating that depletion of infiltrating monocytes could further decrease the BMP4 levels in the injured spinal cord (Fig. 5I,J).

## DISCUSSION

In the present study, we find the following results: first, BMP4 levels in blood and CSF are significantly elevated following an SCI; second, the upregulated BMP4 is particularly accumulated in circulating monocytes and infiltrating MDMs; and third, depletion of circulating monocytes effectively attenuates MDM infiltration and BMP4 accumulation at the lesion site. These results indicate that circulating monocytes provide an important source of BMP4 in the injured spinal cord at the early stage of an SCI.

The role of peripheral BMP4 as an inflammation regulator has gained much attention. Considerable evidence (Helbing et al., 2017; Arnold et al., 2020; Wong et al., 2010; Jo et al., 2006; Pallotta et al., 2017) has revealed that the endothelium- and myocardium-originating BMP4 can impede endothelial barrier function, facilitate leukocyte diapedesis and promote inflammation via directing macrophage polarization towards a pro-inflammatory M1 subtype. By contrast, other research has demonstrated that BMP4 expressed by cancer cells exerts anti-inflammatory actions through favoring the M2 subtype of macrophage (Martínez et al., 2017; Valencia et al., 2019). The discrepancy of BMP4's effect remains to be elucidated. Clinically, circulating BMP4 elevation is detected in patients with cardiac arrest (Arnold et al., 2020; Kercheva et al., 2020), and is relevant to the decreased survival and unfavorable neurological outcome. In addition, our recent clinical research (Zhao et al., 2021) further demonstrated that, during surgery, patients' plasma BMP4 concentration is markedly elevated and is highly correlated with inflammation cytokines, which can be relieved by flurbiprofen (a known cyclooxygenase-II inhibitor), indicating that circulating BMP4 might exert pro-inflammatory action through the cyclooxygenase-II signaling pathway. In the present study, we similarly found that, following CNS injury, the plasma BMP4 levels of patients and SCI rats were markedly elevated compared with those of the uninjured subjects. Based on the evidence above, we propose that these plasma BMP4 elevations play a crucial role in the systemic inflammation regulation after an SCI takes place.

In addition, recent *in vitro* studies have proven that monocytes/macrophages are able to express BMP4 in a lipopolysaccharide-dependent way (Jo et al., 2021), (Wu et al., 2012), and treatment of the exogenous BMP4 promotes monocyte differentiation from the bone marrow CD34^+^ hematopoietic progenitors (Detmer and Walker, 2002), revealing a close relationship between monocyte and BMP4. Here, using an *in vivo* SCI model, we showed that the percentage and MFI of BMP4^+^ circulating monocytes at dpi-3 were markedly higher in SCI rats than in sham rats, indicating that monocytes are an important candidate for the BMP4 upregulation in the circulation. We evaluated CD86 as a marker of activated monocytes based on previous research in which CD86^+^ monocytes displayed strong capability to present antigen (Ugurel et al., 2004) and initiate lymphocyte activation (Pinto et al., 2018), and were highly correlated with higher levels of inflammatory cytokines (Nowak et al., 2019; El Sehmawy et al., 2021). The results showed that BMP4 is more abundantly expressed in the CD86^+^ monocytes. Based on the evidence above, it is reasonable to assume that these MDMs, highly expressing BMP4 and CD86, are activated and play a role in inflammation regulation after an SCI takes place.

BMP4 has been reported to be widely expressed in neurons, astrocytes, oligodendrocytes and ependymal cells in the adult spinal cord (Miyagi et al., 2012). It has been well characterized that, with or without CNS injury, central BMP4 elevation can promote astrocyte activation and inhibit oligodendrocyte differentiation, thus increasing the extent of glial scar formation and demyelination (Wang et al., 2014, 2011; Xiao et al., 2010; Fuller et al., 2007; Hart et al., 2020; Yang et al., 2019). In addition, a growing body of evidence has indicated BMP4 effects on microglial activity and inflammation. Microglia are the residential immune cells in the CNS. Previous research (Miyagi et al., 2012) has demonstrated that multiple types of BMP4-specific receptors are expressed on microglia in the adult spinal cord, indicating a potential role for BMP4 in regulating microglial activity. In addition, our recent study (Liu et al., 2022) demonstrated that intrathecal administration of exogenous BMP4 could induce microglial activation and skew microglia mainly towards the pro-inflammatory subtype, therefore increasing the inflammation in the spinal cord and leading to neuropathic pain. Furthermore, in the process of brain ischemic injury (Samanta et al., 2010; Shin et al., 2014, 2018), antagonizing the BMP4 signaling cascade by noggin application could guide a shift in microglia from iron-storing/pro-inflammatory subtype to iron-released/anti-inflammatory subtype, therefore relieving the inflammation and improving the process of re-myelination. In the present study, we found that BMP4 levels in both injured spinal cord (western blotting) and CSF (ELISA) were sharply elevated. According to the evidence above, we suggest that this central BMP4 upregulation exacerbates the inflammation in the local microenvironment of the spinal cord, which can be detrimental to tissue repair after SCI takes place. Meanwhile, the noggin levels in SCI rats were elevated in the plasma and CSF, which might represent an endogenous protective mechanism aimed at antagonizing the BMP4 signaling cascade. However, no significant change in noggin plasma levels was found in SCI patients. Whether there exists an imbalance between BMP4 and noggin, and the actual role of noggin in the course of an SCI, still need to be elucidated.

Despite the fact that the role of central BMP4 has largely been elucidated, it is still unclear what exactly induces BMP4 upregulation. Previous studies have shown that, in the process of SCI and other CNS disorders, BMP4 is reported to be mainly expressed by most neural cell types (Xiao et al., 2010), astrocytes (Wang et al., 2011) and oligodendrocyte progenitor cells (Sabo et al., 2009; Zhao et al., 2005; Li et al., 2008). However, a growing body of evidence has indicated that microglia/macrophages are another candidate for BMP4 accumulation in the injured CNS. For example, Ara et al. (2008) reported increased BMP4 expression in areas of inflammation, predominantly limited to macrophages, after experimental autoimmune encephalomyelitis. In addition, clinical (Costa et al., 2019) and animal (Harnisch et al., 2019) studies on multiple sclerosis have shown that BMP4 is strongly overexpressed in microglia/macrophages at the inflammatory lesion site, indicating that microglia/macrophage-originated BMP4 may be highly correlated with inflammation in the CNS. In addition to these resident cells in the spinal cord, we also detected abundant MDM infiltration to the spinal cord, which was characterized by high BMP4 expression. This is very much in line with previous literature demonstrating that, after SCI occurs, blood-derived signals [including neutrophils and monocytes (Lee et al., 2011; Akaishi et al., 2018), and fibrinogen (Petersen et al., 2017)] migrate through the disrupted BSCB and play a critical role in controlling neural progenitor cell differentiation.

We further found the following results in the present study: (1) a positive correlation in the levels of BMP4 between blood and CSF, indicating that BMP4 participates in the crosstalk between circulation and CNS when an SCI takes place; (2) the percentage of BMP4^+^ cells in infiltrating MDMs was significantly higher than that in microglia and total microglia/macrophages, and also higher than that in the sham group of rats; (3) the percentage of infiltrating MDMs co-expressing CD86 approached 70%, and the tSNE test further identified that the central BMP4 was mainly colocalized with CD86, which was in agreement with the fact that peripheral BMP4^+^ monocytes highly express CD86; and (4) the depletion of circulating monocytes effectively relieved the SCI-induced upregulation of BMP4 in the injured spinal cord. These results corroborate that circulating monocytes are an important source of BMP4 accumulation in the injured spinal cord.

There are several limitations to the present study. First, we only tested BMP4 expression in circulating monocytes and infiltrating MDMs. The actual role of BMP4 originating from the circulating monocytes needs further investigation. Second, we only evaluated BMP4 expression in the injured spinal cord at 3 days after SCI. Future studies will extend the observation period to investigate BMP4 levels in circulating monocytes and infiltrating MDMs in a longer time frame.

**Fig. 6.**
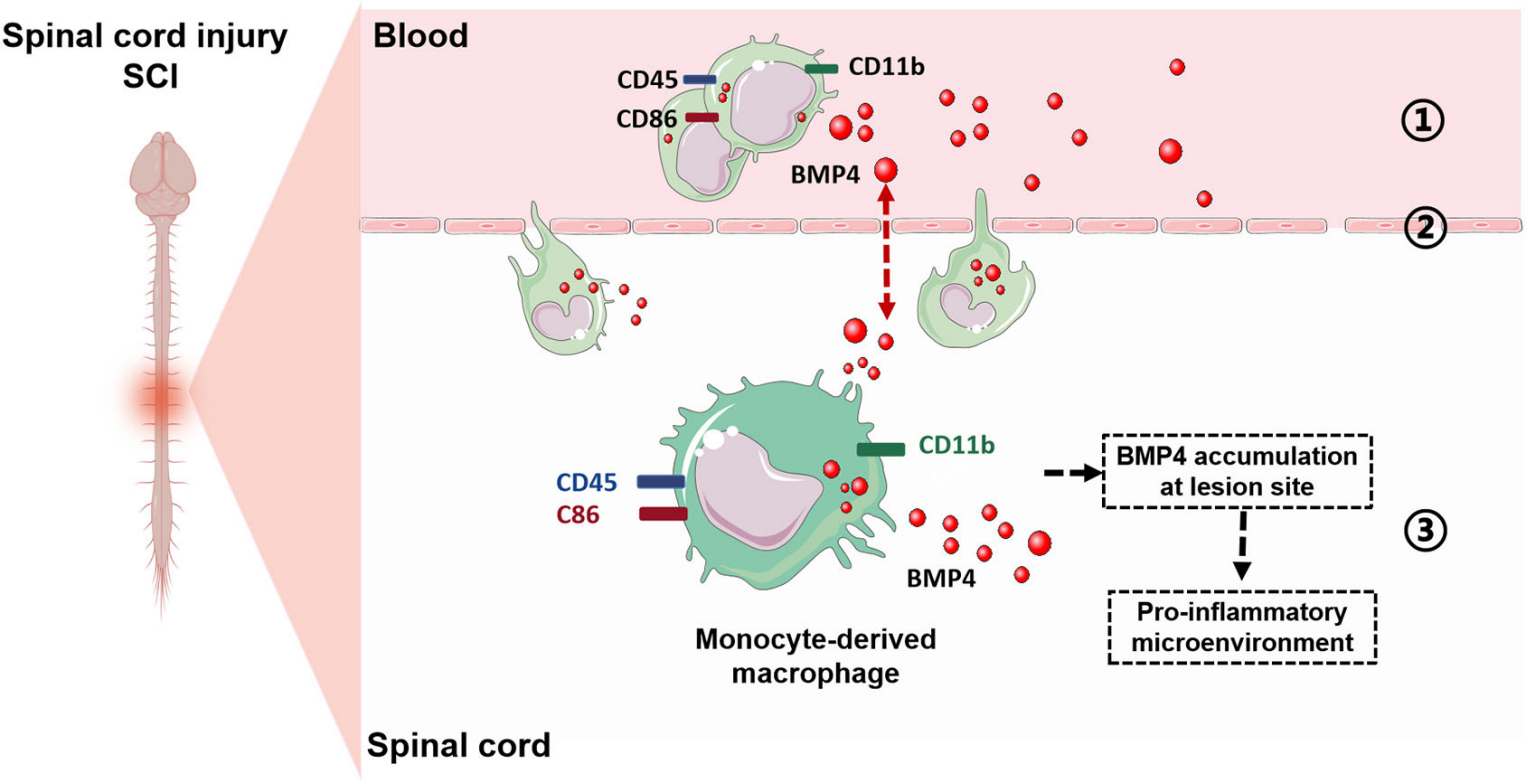
**Possible mechanism of BMP4 upregulation in the process of SCI.** (1) After SCI, BMP4 is highly expressed in the CD86^+^ circulating monocytes; (2) circulating monocytes migrate through the disrupted blood–spinal cord barrier and enhance the infiltration of MDMs in the injured spinal cord; (3) the infiltrating MDMs contribute to the BMP4 accumulation and aggravate the pro-inflammatory microenvironment at the lesion site in the central nervous system.

In summary, our results indicate that, following SCI, circulating monocytes highly express BMP4, migrate through the disrupted BSCB and enhance the infiltration of MDMs in the injured spinal cord. The MDMs, also characterized by upregulated BMP4 expression, contribute to the BMP4 accumulation and aggravate the pro-inflammatory microenvironment at the lesion site in the CNS (Fig. 6).

## MATERIALS AND METHODS

### Patient enrollment

The clinical research involved in the present study was approved by the Second Xiangya Hospital's Institutional Review Board (LYF2020054) and registered prior to patient enrollment at the Chinese Clinical Trial Registry (www.chictr.org.cn) with the registration number of ChiCTR2000039888 (principal investigator, Y.W.; date of registration, 13 September 2020). Written informed consent was obtained from each participant. The trial was conducted in strict accordance with the principles of the Declaration of Helsinki.

From 12 June 2021 to 12 September 2021, 27 patients with acute SC – diagnosed by magnetic resonance imaging or computed tomography – were screened for study eligibility. The severity of injury was assessed with the range from A to E according to the ASIA impairment scale (McDonald and Sadowsky, 2002). Circulating BMP4 levels are closely correlated with obesity, and vascular and metabolic diseases (Jo et al., 2021; García de Vinuesa et al., 2016; Hanna and Frangogiannis, 2019; Baboota et al., 2021; Hoffmann et al., 2017). Hence, the exclusion criteria included the following: (1) patients with hypertension or vascular diseases; (2) patients with obesity, whose body mass index (BMI) was above 30 kg/m^2^; (3) patients with diabetes mellitus; (4) patients receiving long-term steroid treatment (≥3 months); (5) patients with brain trauma, amputation, multiple trauma and other serious injuries. For the control group, 28 healthy volunteers were enrolled. Five milliliters of venous blood from each healthy donor and patient were sampled for further tests.

### Animal grouping

Young adult male Sprague-Dawley rats (8 weeks old, 200-250 g) were purchased from Silake Jingda Experimental Animal Co., Ltd (Changsha, Hunan, China), housed and provided with food and water *ad libitum*. All procedures and practices were approved by the Institutional Animal Care and Use Committee of the Central South University (LYF2021331) and were conducted in accordance with the Guide for the Use and Care of Laboratory Animals of the National Institutes of Health.

Ninety rats were randomly divided into three groups: (1) sham group (*n*=28), rats that underwent a T9 laminectomy without SCI; (2) SCI group (*n*=48), rats that underwent a T9 laminectomy together with SCI induced by falling objects; (3) SCI+CLL group (*n*=8), rats that received two times CLL intra-caudal vein administration, i.e. 3 days before and immediately after SCI intervention (Fig. 5A). The remaining six rats were used to test the efficiency of monocyte depletion using flow cytometry.

### SCI intervention

SCI was conducted according to our previous research (Liu et al., 2021). Briefly, rats in the SCI group and the SCI+CLL group were anesthetized with pentobarbital [30 mg/kg, intraperitoneally (i.p.)] and placed in a prone position. The lamina of T9 was carefully removed to expose the spinal cord while keeping the dura intact. Then, each rat was fixed on a modified Allen's impactor (RWD Life Science, Shanghai, China), with the T8 and T9 spinous processes clamped and stabilized. Subsequently, a 10 g rod was dropped from a height of 5 cm to the exposed T9 spinal cord. Animals that failed to show tail spasms and paralysis of both hindlimbs were excluded from the study. As control, rats in the sham group were only subjected to the T9 laminectomy without SCI intervention. Afterward, the muscles and skin layers were sutured. Penicillin (2000 U/g/day; P8010, Solarbio, Beijing, China) was intra-muscularly injected, and auxiliary urination was performed on the SCI rats three times a day until the end point of the observation period.

### Monocyte depletion

Monocyte depletion was conducted based on Mai et al. (2021). Rats in the SCI+CLL group were injected with CLL (15 ml/kg; 40337ES08, Yeasen Biotechnology, Shanghai, China) via the caudal vein 3 days before and immediately after the SCI to deplete the circulating monocytes. The efficiency of monocyte depletion was evaluated immediately before the SCI intervention by examining the percentage of CD11b^+^ cells in blood using flow cytometry (Fig. 5B).

### BBB scores

The BBB rating scale was employed to assess the behavioral outcomes of rats (*n*=7 for the sham and SCI groups) in the consecutive 2 weeks. The method was introduced in our previous research (Liu et al., 2021). Briefly, rats were placed in a fixed open field and then graded in consideration of several aspects, including joint movement, coordination and physical stability. The evaluation was repeated at a fixed time (15:00-18:00) on dpi-1, dpi-3, dpi-5, dpi-7 and dpi-14 by a researcher who was blinded to the animal grouping.

### ELISA

Venous blood samples for human were drawn and stored in ethylene diamine tetraacetate acid (EDTA)-containing tubes to test the plasma levels of BMP2/4/7 and their physiological antagonist noggin (Zimmerman et al., 1996). After deep anesthesia (100 mg/kg pentobarbital, i.p.), ten rats in the sham group and 30 rats in the SCI group (*n*=10 for each time point at dpi-1, dpi-7 and dpi-14) were euthanized to test the expression of BMP4, noggin and NEFL in the circulation (1 ml collected from the femoral vein) and CSF [200 μl collected from cisterna magna as described previously (Duris et al., 2011)]. All blood samples were centrifuged for 15 min at 3800 ***g*** to obtain the plasma; CSF samples were obtained by centrifugation for 10 min at 3000 rpm to remove the cells and debris (Zimmerman et al., 1996). The levels of BMP2 (HM10833, Bioswamp, Wuhan, China), BMP4 (HM10049, Bioswamp), BMP7 (HM10722, Bioswamp) and noggin (JYM2457Hu, JYM-BIO, Wuhan, China) for human, and the levels of BMP4 (RA20622, Bioswamp), Noggin (JYM12698Ra, JYM-BIO) and NEFL (JYM1167Ra, JYM-BIO) for rats, were tested according to the manufacturers’ instructions.

### Histological section and analysis

Six rats (*n*=3 for the sham and SCI groups at dpi-3) underwent deep anesthesia and were then subjected to trans-cardiac perfusion with 150 ml phosphate buffer solution (PBS) containing 0.1% heparin, followed by 300 ml cold 4% paraformaldehyde. Then, lower thoracic segments of spinal cord at the lesion site were harvested, fixed *in vitro* for 8 h and cryoprotected in 30% (w/v) sucrose. Serial frozen sections of 20 μm thickness, embedded in OCT medium (Sakura Finetek, Torrance, CA, USA), were obtained and stained with H&E.

### Immunofluorescence

The frozen sections obtained above were washed in PBS and rinsed in 0.2% Triton X-100 mixture with 5% donkey serum for 1 h at room temperature. After blocking, each section was incubated with primary antibody rabbit anti-NEUN (1:300; EPR12763, Abcam, Cambridge, UK), followed by Alexa Fluor 594-conjugated AffiniPure Donkey Anti-Rabbit IgG (1:500; 711-585-152, Jackson ImmunoResearch, PA, USA). Next, all sections were washed in PBS and sealed. A fluorescence microscope (Olympus U-HGLGPS, Japan) was used to capture the images, and Image-Pro Plus 6.0 software (Media Cybernetics, Rockville, MD, USA) was used to calculate the intensity in the selected area of each section.

### Western blotting

Nine rats (*n*=3 for the sham, SCI and SCI+CLL groups at dpi-3) were deeply anesthetized and sacrificed. Lower thoracic segments of spinal cord at the lesion site were obtained and homogenized in RIPA lysis buffer (C5029, Bioss, Beijing, China) containing proteinase inhibitor (04693159001, Roche, Mannheim, Germany), followed by centrifugation at 4°C at 15,300  ***g*** for 15 min to collect the supernatants. Then, a bicinchoninic acid method (BCA protein assay kit, CW0014S, CWBIO, Jiangsu, China) was used to test the protein concentrations for each sample. After mixing and boiling with the loading buffer, each sample with 20 μg protein was loaded onto 10% SDS-polyacrylamide gels for electrophoresis, followed by transfer to a polyvinylidene difluoride membrane (Millipore, MA, USA). The membrane was blocked with 5% non-fat dried milk for 1 h at room temperature, and then immunoblotted overnight at 4°C with primary antibodies, including rabbit anti-BMP4 (1:2000; ab39973, Abcam), rabbit anti-CD11b (1:1000; ab133357, Abcam), rabbit anti-CD45 (1:500; ab10558, Abcam), rabbit anti-GFAP (1:3000; Cell Signaling Technology, MA, USA), rabbit anti-CNPase (1:1000; ab227218, Abcam) and rabbit anti-GAPDH (1:6000; Proteintech, Rosemont, IL, USA), followed by incubation with secondary antibody horseradish peroxidase-conjugated goat anti-rabbit IgG (1:6000; Proteintech) for 1 h at room temperature. After washing three times with Tris-buffered saline containing 0.05% Tween 20, each membrane was processed with Luminol Reagent (Millipore, Burlington, MA, USA) and detected using an imaging system (CLiNX, Shanghai, China). All films were then scanned and analyzed with Image-Pro Plus 6.0 software.

### Flow cytometry

Fifteen rats (*n*=5 for the sham, SCI and SCI+CLL groups) were euthanized at dpi-3. To prepare peripheral blood mononuclear cells (PBMCs) for flow cytometric detection, whole blood was collected, and PBMCs were separated by Ficoll (17-5446-02, GE Healthcare, Marlborough, MA, USA) density gradient centrifugation according to our previous research (Shen et al., 2020). To prepare spinal cord mononuclear cells for flow cytometric detection, the segments of thoracic spinal cord at the lesion site (∼0.5 cm length in each sample) were harvested and passed through a 40 μm cell strainer (352350, FALCON, New York City, NY, USA). Then, cells in the suspension were separated from myelin and debris through centrifugation using Percoll density gradient medium (70% and 30%; 17-0891-09, GE Healthcare) as described previously (Li et al., 2021).

Next, isolated cells were washed and resuspended in PBS for further experiments. Live/dead staining (Zombie Aqua™fixable viability kit, 423101, Biolegend, San Diego, CA, USA) was performed at room temperature for 15 min to discriminate the live and dead cells in spinal samples. To analyze the expression of BMP4 on monocytes/macrophages, spinal single cells and PBMCs were blocked with 0.5% bovine serum albumin (BSA) and then stained with following antibodies at 4°C for 30 min: FITC anti-rat CD45 (5 μl/test, 202205, Biolegend), Percp/cyanine5.5 anti-rat CD11b (5 μl/test, 201819, Biolegend) and Alexa Fluor 647 anti-rat CD86 (200314, Biolegend). For intracellular staining, cells were fixed with fixation buffer (00-8222-49, Invitrogen, Carlsbad, CA, USA), and then permeabilized with permeabilization buffer (00-8333-56, Invitrogen). Next, cells were blocked with 0.5% BSA and then incubated with the phycoerythrin (PE)-conjugated anti-BMP4 monoclonal antibody (1:50; bs-1374R-PE, Bioss) or PE-conjugated IgG isotype control (1:50; bs-0295P-PE, Bioss) for 30 min at room temperature.

To evaluate the clearance efficiency of monocytes/macrophages, cells were incubated with FITC anti-rat CD45 or Percp/cyanine5.5 anti-rat CD11b at 4°C for 30 min. After rinsing, stained cells were read on the flow cytometer (Cystek, Fremont, CA, USA), and data were analyzed with FlowJo vX0.7 software.

### Statistical analysis

For parametric variables, data are presented as mean±s.d. BBB scores were analyzed with two-way analysis of variance (ANOVA), followed by Sidak's multiple comparison tests. Protein expression, fluorescence intensity and cytometric data were compared with unpaired two-tailed Student's *t*-test for two groups, and one-way ANOVA for three groups, followed by Sidak's multiple comparison tests at each time point.

For non-parametric variables, including the plasma and CSF concentrations of the above indicators, data are presented as median and interquartile range, and were analyzed with Mann–Whitney *U*-test for two groups and Kruskal–Wallis test followed by Dunn's multiple tests for three groups. Correlations of all mentioned indicators between blood and CSF were analyzed with Spearman's rank correlation coefficient (*r*) based on samples from both groups.

All data were analyzed and charted with SPSS (version 19.0, IBM, Armonk, NY, USA) and Prism software (version 8.0.1, GraphPad, La Jolla, CA, USA). |*R*|>0.3 was identified as having a statistical correlation (Mukaka, 2012), and *P*<0.05 was considered statistically significant.

## Supplementary Material

10.1242/dmm.049856_sup1 Supplementary information
