# Supplementary material for: Infiltrating circulating monocytes provide an important source of BMP4 at the early stage of spinal cord injury
Source: Dis Model Mech. 2023 Jan 18;16(1):dmm049856. doi: 10.1242/dmm.049856 (PMC9884123; doi:10.1242/dmm.049856)
Supplement: Supplementary information [file dmm-16-049856-s1.pdf]

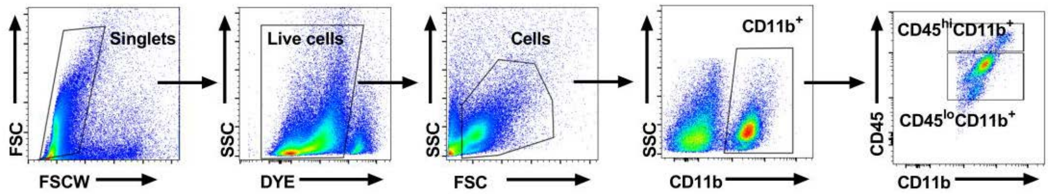

Fig. S1. Representative flow cytometry images showing the steps and gating strategies of monocyte-derived macrophages and macroglia in spinal cord.

Disease Models & Mechanisms • Supplementary information
